# Supplementary material for: Electron Transfer Induced Decomposition in Potassium–Nitroimidazoles Collisions: An Experimental and Theoretical Work
Source: Int J Mol Sci. 2019 Dec 6;20(24):6170. doi: 10.3390/ijms20246170 (PMC6940910; doi:10.3390/ijms20246170)
Supplement: Supplementary File 1 [file ijms-20-06170-s001.pdf]

Supplementary Materials

Electron Transfer Induced Decomposition in Potassium–Nitroimidazoles Collisions: An Experimental and Theoretical Work

M. Mendes[1], G. García[2], M.-C. Bacchus-Montabonel[3*], P. Limão-Vieira[1*]

[1]Atomic and Molecular Collisions Laboratory, CEFITEC, Department of Physics, Universidade NOVA de Lisboa, Campus de Caparica, 2829-516 Caparica, Portugal
[2]Instituto de Física Fundamental, Consejo Superior de Investigaciones Científicas, Serrano 113-bis, 28006 Madrid, Spain
[3]Univ. Lyon, Université Claude Bernard Lyon 1, CNRS, Institut Lumière Matière, 69622 Villeurbanne, France

*Correspondence: bacchus@univ-lyon1.fr; plimaovieira@fct.unl.pt

MP2 geometry of imidazole

```
MP2/VTZ  ENERGY=-225.77625270
  C         0.6276556702       -0.9906757562        0.0000000000
  N        -0.7446199252       -0.9922315319        0.0000000000
  C        -1.0886209374        0.2837489026        0.0000000000
  N         0.0034899157        1.0968081436        0.0000000000
  C         1.1160421810        0.2961704044        0.0000000000
  H        -2.0937939979        0.6676066298        0.0000000000
  H        -0.0041475316        2.1018374677        0.0000000000
  H         2.1131087511        0.6963468682        0.0000000000
  H         1.1913258741       -1.9068711282        0.0000000000
```

MP2 geometry of 2-nitroimidazole

```
MP2/VTZ  ENERGY=-429.99079192
  C         1.1495965581        0.2923270228        0.0525798549
  C         0.6510681417       -0.9949251256       -0.0356569248
  N        -0.7114442985       -0.9907948905       -0.1481639775
  C        -1.0198636759        0.2886434699       -0.1278731008
  N         0.0520985048        1.1044904026       -0.0097103715
  N        -2.3362894136        0.8607993674       -0.2153358714
  H        -0.0019850052        2.1104114959        0.0244893917
  H         2.1469322378        0.6802770720        0.1511726539
  H         1.2066736546       -1.9159428178       -0.0207421709
  O        -2.3816041437        2.0954322319       -0.1732933885
  O        -3.2903045604        0.1018857714       -0.3204950950
```

MP2 geometry of 4-nitroimidazole

```
MP2/VTZ  ENERGY=-429.99129456
  C         1.1300154843        0.2878354547        0.0562114534
  C         0.5888329181       -0.9496134371       -0.2126401760
  N        -0.7623782681       -0.9291051948       -0.2948866307
  C        -1.0739621748        0.3367684858       -0.0746160671
  N         0.0347728392        1.1000859510        0.1411406514
```

H	-2.0697562195	0.7434492243	-0.0614253023
H	0.0485857913	2.0891430282	0.3277851806
H	2.1410778772	0.6223421493	0.1855688138
N	1.3543426719	-2.1607393353	-0.3932582122
O	2.5763687984	-2.0422170259	-0.2940291881
O	0.7462714601	-3.1978069188	-0.6280645572

MP2 geometry of 1-methyl-4-nitroimidazole

MP2/VTZ ENERGY=-469.20988603

C	1.0053751239	0.3251193371	0.1715189994
C	0.5437529781	-0.9235938967	-0.1902585480
N	-0.8051248435	-1.0071531324	-0.1859097755
C	-1.1887493611	0.2052899262	0.1845953065
N	-0.1340922171	1.0398846220	0.4102203317
H	-2.2079164588	0.5320525364	0.3043800633
C	-0.2081020948	2.4274793634	0.8268409649
H	1.9951225173	0.7293514112	0.2707826349
N	1.3854853023	-2.0411599077	-0.5450448059
O	2.5993475091	-1.8314032638	-0.5102326444
O	0.8455249047	-3.0979259929	-0.8501692090
H	0.2788943091	2.5572864248	1.7895943639
H	0.2672305988	3.0666704973	0.0876262253
H	-1.2543358511	2.7033371651	0.9162737475

MP2 geometry of 1-methyl-5-nitroimidazole

MP2/VTZ ENERGY=-469.20752236

C	0.9316098962	0.3119234837	0.1542582996
C	0.5247132317	-0.9653796136	-0.1830635355
N	-0.8324415506	-1.0314803723	-0.1505534015
C	-1.2299216307	0.1847063430	0.2000982001
N	-0.1957669310	1.0475644750	0.3990843223
H	-2.2511799886	0.5030967007	0.3260173221
C	-0.3400054931	2.4418958553	0.7920564374
N	2.2569204515	0.8331529702	0.2480906446
H	1.1522071543	-1.8005999107	-0.4367045903
O	2.3903188635	2.0179095607	0.5659689839
O	3.1715173352	0.0528009771	0.0000221670
H	0.1561751250	2.6196764163	1.7397809953
H	0.0925822873	3.0923259561	0.0399385466
H	-1.4043163340	2.6363007617	0.8860269698
